# The Impact of Consuming Probiotics and Following a Vegetarian Diet on the Outcomes of Acne

**DOI:** 10.7759/cureus.51563

**Published:** 2024-01-03

**Authors:** Abdullah Alyoussef

**Affiliations:** 1 Internal Medicine, Faculty of Medicine, University of Tabuk, Tabuk, SAU

**Keywords:** vegetarian diet, topical acne treatment, immune response, probiotics, acne

## Abstract

Acne vulgaris is a common skin condition that affects millions of people worldwide. While the exact cause of acne is not fully understood, it is believed to be influenced by various factors such as the skin microbiome, host immunity, hormones, genetics, and possibly diet. There are several treatment options for acne, including antibiotics and vitamin A derivatives (retinoids). However, these treatments can have side effects, such as dryness, redness, and peeling of the skin. The relationship between diet and acne remains somewhat controversial. Studies have found that Western societies have a higher incidence of acne than non-Western societies, which is believed to be due to dietary differences. Several experiments were conducted to target the skin microbiome and treat acne with the hope of using probiotics orally or topically to regulate the immune response and reduce inflammation in acne. In addition, studies have shown that a plant-based diet can benefit individuals with acne. Avoiding dairy consumption is one of the most effective dietary changes for reducing acne. As part of our investigation, we conducted a review to determine the effect of probiotics and vegetarian diets on acne.

## Introduction and background

Acne vulgaris is a common skin condition that affects millions of people worldwide. It involves the hair follicles and oil glands, causing inflammation. It can result in the formation of comedones (blackheads and whiteheads), papules (small red bumps), pustules (pus-filled blisters), or abscesses on the face, neck, trunk, and arms [1]. While it is most commonly observed in teenagers and young adults, it can affect people of all ages. Studies have shown that up to 85% of individuals between 12 and 24 years of age experience some form of acne. Among these, around 15% to 20% experience moderate-to-severe acne that can lead to permanent scarring if left untreated [2]. Proper management and timely treatment of acne can help prevent the development of scarring and improve the overall appearance and health of the skin.

While the exact cause of acne is not fully understood, it is believed to be influenced by various factors that interact with one another. These factors include the skin microbiome, host immunity, hormones, genetics, and possibly diet [3]. Acne lesions are typically classified into two categories: inflammatory and noninflammatory. Inflammatory lesions include papules, pustules, and abscesses, while noninflammatory lesions are known as comedones. Microcomedones are considered to be the initial form of acne and are formed when the skin cells lining the hair follicle become detached, leading to a small, hyperkeratotic plug in the follicular infundibulum. The development of microcomedones into acne lesions is believed to occur due to the interaction of several factors. Follicular hyperkeratinization, increased sebum production, colonization of skin bacteria, and inflammation are all thought to play a role in the formation of acne lesions [4]. Acne can manifest in various forms, such as mild papular lesions, moderate papular-pustular lesions, and severe nodulocystic lesions. These lesions can be painful and unsightly, affecting a person's self-image and confidence. In addition, acne can leave scars after the acute phase, which can further harm a person's self-esteem and lead to social isolation [5].

There are several treatment options for acne, including oral and topical antibiotics, oral contraceptives, oral corticosteroids such as prednisolone, and vitamin A derivatives (retinoids). However, these treatments can have side effects, such as dryness, redness, and peeling of the skin. These side effects can be uncomfortable and reduce patients' adherence to the treatment regimen. Therefore, monitoring patients for side effects and adjusting their treatment plan accordingly is essential to achieve the best possible outcomes [6].

## Review

Role of nutrition in acne

Dietary manipulation has been an area of considerable interest in the etiology, treatment, and prevention of dermatologic diseases. The relationship between diet and acne remains somewhat controversial. Acne has been linked to diet. Studies have found that Western societies have a much higher incidence of acne than non-Western societies, which is believed to be due to dietary differences. Western diets often lack long-chain omega-3 fatty acids but contain high amounts of refined carbohydrates. The imbalance between omega-6 and omega-3 fatty acids is thought to play a significant role in the development of acne [7].

While pharmacological treatments can be effective, recent studies have shown that combining these treatments with a healthy diet and vitamin supplements may further improve acne lesions. In fact, research has shown that the microbiome - the collection of microorganisms living within and on our bodies - plays a significant role in this context. Specifically, scientists have been studying the role of the microbiome in both the gut and on the skin, and how it can influence the development and severity of acne [8]. This new understanding could lead to more effective and holistic treatments for acne patients.

Studies have shown that milk consumption and high glycemic loads, which are diets that cause a rapid and large increase in blood glucose levels, are both independently associated with increased levels of serum insulin-like growth factor-1 (IGF-1). This suggests a possible mechanism for how these dietary factors may contribute to the development of certain diseases, as IGF-1 has been linked to an increased risk of cancer and other health issues [9].

Acne is a prevalent disease in Western societies that is closely related to the Western diet. Interestingly, populations that follow a Paleolithic diet, which excludes sugar, grains, and dairy protein, such as the Kitava islanders, do not suffer from acne or other epidemic diseases of civilization. They exhibit low basal insulin levels compared to age-matched Europeans [10]. A randomized placebo-controlled Australian trial found that reducing the glycemic load in the diet improved the clinical symptoms of acne, as well as the rate of sebum excretion and free androgen index in male acne patients between 15 and 25 years of age [11].

A study conducted in South Korea has provided further evidence of the link between food composition and acne. The study included a total of 1,285 participants, comprising 783 patients with acne and 502 healthy control subjects. The findings revealed that the control group consumed more vegetables and fish, while patients with acne reported a higher intake of instant noodles, junk food, carbonated drinks, snacks, processed cheeses, pork, chicken, nuts, and seaweed [12]. The study suggests that the Western diet, which is typically high in glycemic load, fat, and dairy and meat consumption, plays a significant role in exacerbating acne. Surprisingly, almost half of the male and female acne patients reported that their condition was worsened by their food intake.

Probiotics and acne

The human skin is not just a physical barrier that protects our bodies from external factors but is also home to a diverse community of microorganisms known as the skin microbiome. These microorganisms work together to maintain the natural balance and integrity of the skin. In the past, several experiments were conducted to target the skin microbiome and treat acne. One such experiment involved using *Lactobacillus bulgaricus*, which reduced seborrhea and the overproduction of oil on the skin [13]. Recent studies have suggested that probiotics can be used topically to regulate the immune response, reduce inflammation, and promote the production of anti-inflammatory cytokines such as interleukin-10. Specifically, lactobacilli, a group of probiotics, have been found to have an antibacterial effect on acne. However, it is crucial to note that each species of bacteria has unique characteristics, and moving it to a different environment can alter its function [14].

Numerous studies have shown the importance of the skin microbiome in acne patients and how altering it can lead to significant clinical improvements. In a Russian study involving 144 patients, those with acne were found to have differences in their intestinal bacterial flora. When treated with probiotics, these patients saw a reduction in the duration of traditional pharmacological treatments. Therefore, it is essential to understand the role of the skin microbiome in maintaining healthy skin and how probiotics, specifically lactobacilli, can be used to regulate the immune response and combat acne [15].

Probiotics such as *Lactobacillus acidophilus* and *Bifidobacterium bifidum *have been found to improve skin barrier function and reduce the severity of acne in clinical trials. Some studies have also shown that oral probiotics can reduce the number of acne lesions and improve skin quality [16,17]. However, more research is needed to determine the best strains and dosages of probiotics for acne treatment.

Topical probiotics

Topical probiotic treatment is a promising option for treating acne, as it is generally regarded as safe and well-tolerated, with fewer side effects than conventional therapy. However, there is a dearth of clinical evidence evaluating the efficacy of probiotics in topical pharmaceutical formulations for acne treatment. While there are a growing number of commercial products that claim to offer beneficial effects for acne, based on the inclusion of probiotics, the current evidence supporting these claims is insufficient, and more research is needed to determine their true effectiveness [18].

Through laboratory studies, researchers have discovered that the bacteria *Staphylococcus epidermidis* and *Enterococcus faecalis* are capable of directly inhibiting the growth of acne by producing antibacterial proteins, including a bacteriocin-like inhibitory substance [19]. Another study conducted by Wang revealed that *S. epidermidis *could ferment glycerol and produce short-chain fatty acids, which have also been found to have antibacterial properties that can inhibit the growth of acne [20].

A clinical trial was conducted by Kang et al. to evaluate the effectiveness of a concentrated powder lotion derived from *E. faecalis* culture's supernatant on acne patients. The trial included 70 patients with acne and lasted for eight weeks. The trial was designed as double-blind and randomized, where the patients were treated with either the concentrated powder lotion or a placebo lotion. The results showed that the patients who received the concentrated powder lotion had a significant reduction in inflammatory lesions compared to those who received the placebo lotion [21].

In 2016, a study was conducted to investigate the effects of substituting glycerol with sucrose on the fermentation of *S. epidermidis* without affecting acne. The study involved injecting acne and *S. epidermidis* into the ears of murine models, to which either sucrose or phosphate-buffered saline was added. The results of the study showed that the growth of the pathogen was significantly reduced in the group with sucrose, and there was a marked decrease in the production of macrophage inflammatory protein-2 (MIP-2). These findings suggest that sucrose has the potential to selectively promote the fermentation of *S. epidermidis *while inhibiting the growth of acne [22]. Another study has shown that *Streptococcus salivarius *has the potential to inhibit the growth of acnes in vitro. This is achieved through the production of bacteriocin-like inhibitory substances, which also can modulate the inflammatory response [23].

In a clinical trial conducted in 2017, 358 adult patients with mild or moderate acne were administered the ammonia-oxidizing bacteria known as *Nitrosomonas eutropha*. The trial lasted for 12 weeks, and the results showed a significant decrease in the overall severity of the condition. This was attributed to the ability of the ammonia-oxidizing bacteria to convert ammonia to nitrite, which has antibacterial properties that can be useful in treating skin conditions. Moreover, the trial also showed a noticeable trend in the reduction of inflammatory lesions when compared to the control group. This is because the ammonia-oxidizing bacteria help regulate inflammatory and vasodilation processes by converting ammonia to nitric oxide [8].

In a study focused on the benefits of topical probiotics, researchers experimented with using polysulfone microtube array membrane (MTAM) to enclose *S. epidermidis*, a type of bacterium. The study involved exposing murine models to acne and then applying a topical formulation of membranes that contained enclosed *S. epidermidis* and glycerol. The results showed that this formulation was highly effective in increasing the fermentation activity of glycerol and reducing the levels of acne and inflammation in the skin. The success of the formulation was attributed to the action of succinic acid, which is produced by the enclosed *S. epidermidis* and has been found to have anti-inflammatory properties [24].

In 2022, Sathikulpakdee et al. conducted a randomized clinical trial to assess the efficacy of a probiotic-based lotion in comparison to a conventional 2.5% benzoyl peroxide lotion for the treatment of mild-to-moderate acne in 104 patients over a duration of four weeks. The probiotic-based lotion was derived from the supernatant of a culture of *Lactobacillus paracasei*, a bacterial strain that has been shown to exhibit antimicrobial activity against acne. Both lotions resulted in a significant reduction in the number of acne lesions and erythema index. The study concluded that the probiotic-based lotion derived from *Lactobacillus paracasei* is a safe and effective alternative to the commonly used 2.5% benzoyl peroxide lotion for the treatment of mild-to-moderate acne [25].

Oral probiotics

In the quest to find effective treatments, various studies have been conducted to determine the effects of probiotics on acne, both orally and topically. One clinical study aimed to examine the effects of fermented milk enriched with 200 mg lactoferrin daily for 12 weeks. The study involved patients who were given milk, and the results showed a significant reduction in the number of inflammatory lesions in 38.6% of the patients [26]. This study has shown that lactoferrin positively impacts acne and could be used as a potential treatment.

Another study was conducted on 45 women divided into three groups: one treated with probiotics only, one treated with minocycline only, and one treated with both. The probiotics used in the study were a mix of *Lactobacillus acidophilus* (NAS super-strain), *Lactobacillus delbrueckii* subspecies bulgaricus (LB51 super-strain), and Bifidobacterium bifidum. The results showed a significant reduction in the total number of lesions and in inflamed lesions in the group treated with minocycline and probiotics at weeks 8 and 12. All groups showed clinical improvement, with the combination of minocycline and probiotics achieving significant improvement as early as week 4. This study highlighted the effectiveness of using probiotics with traditional treatments such as minocycline. Moreover, the probiotic-only group showed a more significant reduction in non-inflamed lesions than the minocycline-only group at weeks 4, 8, and 12. This demonstrates that probiotics have the potential to be an effective stand-alone treatment for acne and could be considered an alternative to traditional treatments [27]. Overall, these studies have shown that probiotics can play a significant role in the treatment of acne, and further research is needed to determine the most effective ways to use them [28].

In a study conducted by Fabbrocini et al. back in 2016, it was found that taking *Lactobacillus rhamnosu*s SP1 orally for 12 weeks resulted in a significant decrease in the expression of IGF-1 and forkhead box O1 (*FOXO1*) in the skin. The research was conducted on a group of patients; some were treated with *Lactobacillus rhamnosus*, while others were given a placebo. At the end of the 12-week trial period, it was found that the group of patients who were treated with *Lactobacillus rhamnosus *showed a 32% increase in the genes that code for *FOXO1*. At the same time, there were no statistically significant differences observed in the placebo group. These findings further prove the potential health benefits of using Lactobacillus rhamnosus SP1 in promoting healthy skin [29].

In 2022, Mosaico et al. conducted a study that involved two teenage patients, a 14-year-old girl and a 15-year-old boy, who were undergoing fixed orthodontic treatment for acne vulgaris. The researchers observed that both patients exhibited signs of gingivitis with high scores of full mouth plaque and bleeding on probing. To address this issue, the patients were first treated with professional oral hygiene sessions and scaling and root planing procedures. Afterward, the patients were administered a probiotic formulation that contained *Lactobacillus reuteri*, which is known to have beneficial effects on oral health. After four weeks, the researchers conducted a follow-up, which showed significant clinical improvement in both patients for gum hypertrophy and skin acne vulgaris [30].

In a rigorous clinical trial involving 20 adult subjects, the efficacy of the oral probiotic *Lactobacillus rhamnosus *SP1 was evaluated through a double-blind, placebo-controlled, randomized study. The study aimed to investigate the effect of the probiotic on gene expression and clinical outcomes. The results showed that after 12 weeks of treatment, there was a significant reduction in the expression of the IGF-1 gene by 32%. Additionally, the study found a considerable increase in the FOXO1 gene by 65% in the group receiving the probiotic treatment. Notably, no significant changes were reported in the placebo group. Moreover, the patients who received the probiotic treatment demonstrated significant clinical improvement, such as reduced bloating and abdominal pain, improved bowel movement, and overall well-being [29].

In a double-blind clinical trial, a group of men with mild-to-moderate acne were administered an oral supplement containing probiotics, biotin, vitamin E, zinc, nicotinamide, β-sitosterol, and *Boswellia serrata* extract. Over a period of 12 weeks, the patients were regularly monitored for any changes in their skin condition. At the end of the trial, the results showed significant clinical improvement based on the reduction of the Global Acne Grading System (GAGS) score, indicating a decrease in the severity of acne symptoms [31].

In 2022, Rinaldi et al. conducted a clinical trial to investigate the efficacy of a combination of three probiotic strains - *Bifidobacterium breve*, *Lacticaseibacillus casei*, and *Ligilactobacillus salivarius*, in combination with a botanical extract of *Solanum melongena* and *Echinacea* - in treating mild-to-moderate acne. The trial was conducted over a period of 8 weeks, with 114 participants enrolled in the study. The results of the trial were promising, demonstrating a significant reduction in acne lesions, rate of desquamation, rate of sebum secretion, and presence of acne in the patients who received the probiotic mixture and the botanical extract, as well as the mixture of both, compared to those who received a placebo treatment. The probiotic mix plus the botanical extract was found to be the most effective treatment option, which suggests that the combination could be a safe and effective alternative for treating mild to moderate acne [32].

Vegan and vegetarian diets

People who opt for a vegetarian diet avoid eating meat or animal flesh, which includes fish, poultry, seafood, and any food items containing them. However, there are different types of vegetarianism with varying dietary restrictions. Ovolactovegetarianism is one variation that allows the consumption of dairy products and eggs in addition to plant-based foods. Lactovegetarianism is another variation that permits the consumption of dairy products, but not eggs or other animal-based foods. Finally, vegans follow the strictest form of vegetarianism and avoid all animal-derived foods, including dairy, eggs, and even honey [33]. A plant-based diet has become popular due to reasons such as health benefits, environmental impact, and animal welfare concerns. Many global nutrition organizations have recommended a well-organized plant-based diet as it helps to protect against various chronic ailments such as hypertension, diabetes, cardiovascular diseases, and obesity.

While some dermatologists acknowledge the potential benefits of a vegan diet in treating or preventing skin diseases, others remain unconvinced. There is a scarcity of literature on the subject, and only a few studies have solely concentrated on the vegan community. Certain research has brought to attention that individuals who follow a vegan diet but do not maintain a well-balanced one may suffer from nutritional deficiencies, such as vitamin B12, vitamin D, iron, zinc, calcium, and omega-3. These essential nutrients are crucial for maintaining optimal health. These studies, however, may not accurately represent vegans as a whole but rather a population with nutritional deficits. It is crucial to follow a well-planned vegan diet that meets all necessary nutritional requirements to avoid any potential health risks. A well-balanced vegan diet should include a variety of plant-based protein sources, such as beans, lentils, tofu, and tempeh, along with a wide range of fruits, vegetables, whole grains, nuts, and seeds. It is necessary for vegans to have an adequate intake of nutrients such as vitamin B12, calcium, and iron, and, in some cases, supplements may be required to meet these requirements [34].

Vegetarian diet and acne

Acne is a multifactorial skin condition that can be influenced by various factors, such as genetics, hormones, inflammation, and environmental factors. The role of diet in the pathogenesis of acne has been extensively studied because it can affect many underlying mechanisms. Studies have shown that a plant-based diet can benefit individuals with acne. Avoiding dairy consumption is one of the most effective dietary changes for reducing acne. Cow's milk contains casein, a protein that increases IGF-1 levels [35]. This hormone promotes the overproduction of sebum, which can contribute to acne development. Additionally, whey proteins have insulin-like effects that can elevate IGF-1 levels and worsen acne flares in patients who consume high amounts of whey protein, such as athletes or bodybuilders.

Studies have demonstrated that the consumption of whole or skimmed cow’s milk can worsen the symptoms of acne. Skimmed milk, in particular, contains a higher concentration of hormonal components or other bioactive molecules, such as steroids, α-lactalbumin, growth factor stimulating hormones, and IGF-1, which can exacerbate the condition [36]. It is difficult to systematically evaluate the vast heterogeneity of processed dairy products, but some cow's milk-containing foods, such as ice cream, have been associated with acne.

Research has indicated that the consumption of soy-based products can be beneficial in reducing the likelihood of developing acne. Soy contains isoflavones and phytoestrogens, which have been found to counteract the production of sebum that is triggered by androgens, thus preventing acne breakouts. A study conducted on individuals with acne showed that taking 160 mg of isoflavones each day for a period of 12 weeks led to a significant decrease in the number of acne lesions. Additionally, soy proteins are more favorable than cow's milk proteins in terms of body composition. Soy proteins have been shown to decrease visceral fat accumulation, which is the fat that builds up around organs and can cause various health issues. A decrease in visceral fat could also lead to an improvement in acne [37]. Therefore, incorporating soy-based products into your diet can help improve body composition and prevent acne breakouts.

Consuming saturated fats, which are commonly found in fried foods and animal-derived products such as meat, cheeses, and butter, has been shown to increase IGF-1 levels. High levels of IGF-1 have been associated with an increased risk of certain cancers, including breast and prostate cancer. Additionally, diets that include protein sources such as cow's milk or meat tend to have higher levels of leucine, which activates the same pathway as IGF-1 and can cause inflammation. This inflammation has been linked to chronic diseases such as heart disease, stroke, and diabetes. In contrast, vegetable oils contain polyunsaturated fatty acids such as gamma-linolenic acid, which has anti-inflammatory properties that can help reduce inflammation. This is an essential factor in preventing and managing chronic diseases [38]. Based on the above scientific research, it is evident that a well-balanced diet can have a positive impact on overall body health and effectively prevent chronic diseases. Furthermore, it has been observed that the benefits of a healthy diet extend to skin health as well. In fact, a nutritious diet can play a crucial role in preventing a commonly occurring skin condition, acne.

Incorporating additional fruits and vegetables into your diet can provide immense health benefits. These foods are abundant in anti-inflammatory and antioxidant properties that can safeguard and enhance your health. According to research, a diet centered around plant-based foods and supplemented with vegan polyphenols may help diminish acne blemishes through microbiota adjustment, inflammation reduction, insulin resistance, and hormonal balance [39].

## Conclusions

Acne vulgaris is a disease that is influenced by many factors, including genetics, metabolism, and hormones. The presence of acne is associated with both the skin and gut microbiota. A diet high in fat or with a high glycemic index can increase intestinal permeability and exacerbate acne. To prevent or improve acne, the intestinal microbiota can be modulated. There is research on the effects of probiotics on acne, although some studies have suggested that they may be beneficial. A plant-based diet that excludes dairy products can also be beneficial for individuals with acne by reducing sebum production, hyperkeratinization of the pilosebaceous follicles, and inflammation, all of which are involved in the development of acne.
